# Evolution of generalist resistance to herbicide mixtures reveals a trade-off in resistance management

**DOI:** 10.1038/s41467-020-16896-0

**Published:** 2020-06-18

**Authors:** David Comont, Claudia Lowe, Richard Hull, Laura Crook, Helen L. Hicks, Nawaporn Onkokesung, Roland Beffa, Dylan Z. Childs, Robert Edwards, Robert P. Freckleton, Paul Neve

**Affiliations:** 1 Department of Biointeractions and Crop Protection, Rothamsted Research, Harpenden, Hertfordshire AL5 2JQ UK; 2 Department of Animal and Plant Sciences, University of Sheffield, South Yorkshire, S10 2TN UK; 3 School of Animal, Rural and Environmental Sciences, Nottingham Trent University, Southwell, NG25 0QF UK; 4 School of Natural and Environmental Sciences, Newcastle University, Newcastle, NE1 7RU UK; 5 Bayer Crop Science, Weed Resistance Research, 65926 Frankfurt, Germany; 6 Agriculture & Horticulture Development Board, Stoneleigh Park, Kenilworth, CV8 2TL UK

**Keywords:** Agroecology, Evolutionary theory, Plant evolution

## Abstract

Intense selection by pesticides and antibiotics has resulted in a global epidemic of evolved resistance. In agriculture and medicine, using mixtures of compounds from different classes is widely accepted as optimal resistance management. However, this strategy may promote the evolution of more generalist resistance mechanisms. Here we test this hypothesis at a national scale in an economically important agricultural weed: blackgrass (*Alopecurus myosuroides)*, for which herbicide resistance is a major economic issue. Our results reveal that greater use of herbicide mixtures is associated with lower levels of specialist resistance mechanisms, but higher levels of a generalist mechanism implicated in enhanced metabolism of herbicides with diverse modes of action. Our results indicate a potential evolutionary trade-off in resistance management, whereby attempts to reduce selection for specialist resistance traits may promote the evolution of generalist resistance. We contend that where specialist and generalist resistance mechanisms co-occur, similar trade-offs will be evident, calling into question the ubiquity of resistance management based on mixtures and combination therapies.

## Abstract (web summary)

Mixtures of antibiotics or pesticides can help reduce the evolution of resistance to individual compounds. Here, Comont et al. show that in blackgrass, an important agricultural weed, herbicide mixtures do reduce specialized resistance but instead can select for a generalized resistance mechanism.

## Introduction

The provision of adequate food and the effective treatment of infectious diseases are prerequisites for a healthy human society. A range of chemical agents (pesticides, antimicrobials and anti-retrovirals) has become critical tool for combatting an array of pathogens, pests, weeds and diseases that impact food production and human health. The successful and widespread use of these chemicals has, however, resulted in the rapid and repeated evolution of resistance across taxa^1^. Resistance to antimicrobials has been described as a catastrophic threat to human health and well-being^2^, while fungicide, insecticide and herbicide resistance now seriously compromise food security^3–5^. Developing strategies to mitigate the evolution of resistance has therefore become critical in both agriculture and healthcare.

For many pest and pathogen targets, multiple chemicals with different modes of action are available for use, and a key question is how to best deploy these to slow or prevent the evolution of resistance^1^. Diversifying selection pressure by employing cycling, sequences or mixtures of chemicals with different modes of action is recognised as a key component of resistance management^1,6–12^. In particular, modelling studies across taxa have established the generality of mixtures for effective resistance management^10,11,13–15^ and empirical studies have confirmed this for fungicide^16^, herbicide^17^, insecticide^18^ and antibiotic^19^ resistance. However, the success of mixture strategies rests on the assumption that resistance traits are ‘specialist’ adaptations that do not provide general cross-resistance across modes of action^1^. Cases of target-site resistance (TSR), where the structure and/or expression of the chemical’s biological target are altered, conform to this model^20–23^. Nevertheless, it is now clear that non-target-site resistance mechanisms (NTSR) are also widespread. These mechanisms involve the regulated expression of multiple genes encoding proteins involved in chemical detoxification, transport, efflux and sequestration^24–29^, and can cause simultaneous resistance to multiple chemicals with differing modes of action^30–34^. Consequently, NTSR can provide a more ‘generalist’ resistance^24,25,35,36^.

General theories of ecological specialisation predict that the degree of environmental heterogeneity determines the evolution and maintenance of specialist and generalist strategies^37^. Heterogeneous selective environments can constrain the evolution of specialist adaptations, whilst promoting the evolution of species with more generalist traits^38–41^. In relation to pesticide resistance, repeated application of the same mode of action (MOA) creates a homogenous selective environment, whereas deploying a diversity of modes of action provides heterogeneous selection^1^. In this context, the use of mixtures would be considered a heterogeneous selective environment, predicted to favour the evolution of generalist resistance mechanisms such as NTSR. Where this found to be the case, it could call into question the ubiquity of widely accepted resistance management principles.

Here, we use blackgrass (*Alopecurus myosuroides*), an economically important agricultural weed species^42^, to investigate the evolution of specialist and generalist herbicide resistance mechanisms under varying selection regimes. Blackgrass has evolved resistance to seven different herbicide modes of action^43^, endowed by both specific target-site mutations^44^ and more generalist herbicide detoxification mechanisms^31,45^. We employ a national-scale epidemiological approach, e.g.,^46,47^ to assess variation in herbicide resistance and cross-resistance, the relative importance of specialist and generalist resistance mechanisms, and the response of these mechanisms to different historical herbicide selection regimes. Our results demonstrate a clear epidemiological link between the use of herbicide mixtures and the resistance mechanism that is selected in populations, with more intensive use of herbicide mixtures associated with lower levels of specialist, TSR resistance, but higher levels of the more generalist NTSR mechanism.

## Results

### Herbicide mixtures do not consistently reduce selection for herbicide resistance

**Fig. 1:**
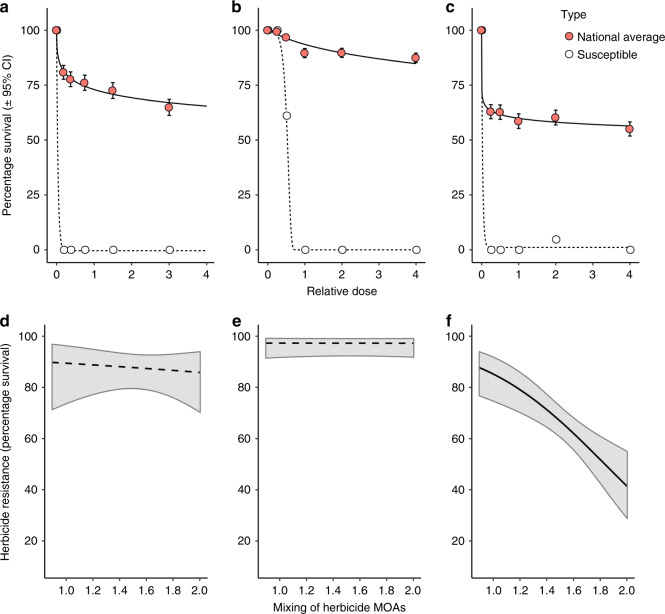
Assessment of phenotypic herbicide resistance in UK *A. myosuroides* populations. The fitted relationships between herbicide dose and plant survival are shown for (**a**) the ‘SU’ herbicide mesosulfuron, (**b**) the ‘Fop’ fenoxaprop and (**c**) the ‘Dim’ cycloxydim. Red filled circles and solid lines represent the mean response across all 132 field-collected populations, providing an estimate of the national level of resistance to these herbicides. For comparison, dotted lines and unfilled circles show the response in a herbicide susceptible standard population. Error bars show the 95% confidence interval about the mean. **d**–**f** show the predicted relationship between the historical intensity of herbicide mixtures applied to field-collected populations and population-level resistance (percentage survival following glasshouse resistance assays) to the SU, Fop and Dim herbicides, respectively. Fitted lines show the mean predicted relationship following mixed model analysis (see Supplementary Table 1), with shaded regions representing the 95% confidence interval. A solid black line is used for a significant relationship (**f**) (*p* ≤ 0.001), while dashed lines are used where the relationship was non-significant (**d**, **e**). Source data for panels (**a**–**c**) are provided as a Source Data file. Experiments were conducted once for each herbicide, screening 108 plants per population over six doses.

Plant phenotyping assays for 132 UK blackgrass populations identified high levels of evolved resistance to three herbicides: the sulfonylurea (SU) herbicide mesosulfuron (‘SU’, >75% survival at field-relevant dose), the aryloxyphenoxypropionate herbicide fenoxaprop-P-ethyl (‘Fop’, >90% survival), and the cyclohexanedione-oxime herbicide cycloxydim (‘Dim’, >60% survival), while all reference susceptible populations were completely controlled (Fig. 1a–c, Supplementary Fig. 1). Seventy-nine per cent of populations showed resistance (≥20% survival) to all three herbicides, suggesting widespread presence of cross- or multiple-herbicide resistance. The historical intensity of the use of a particular class of herbicide (SU, Fop or Dim) was positively associated with phenotypic resistance to that herbicide class, clearly establishing that the resistance epidemic was driven by herbicide use (Supplementary Table 1). The historical intensity of the use of herbicide mixtures was negatively associated with ‘Dim’ resistance, but phenotypic ‘Fop’ and ‘SU’ resistance were not associated with either herbicide mixture intensity or herbicide diversity (Fig. 1d–f, Supplementary Table 1). Therefore, at the whole plant phenotypic level, it appears that resistance to the ‘Dim’ herbicide was mitigated by herbicide mixtures, whereas resistance to ‘Fop’ and ‘SU’ herbicides was unaffected.

### High frequencies of target-site mutations only partially account for observed resistance

**Fig. 2:**
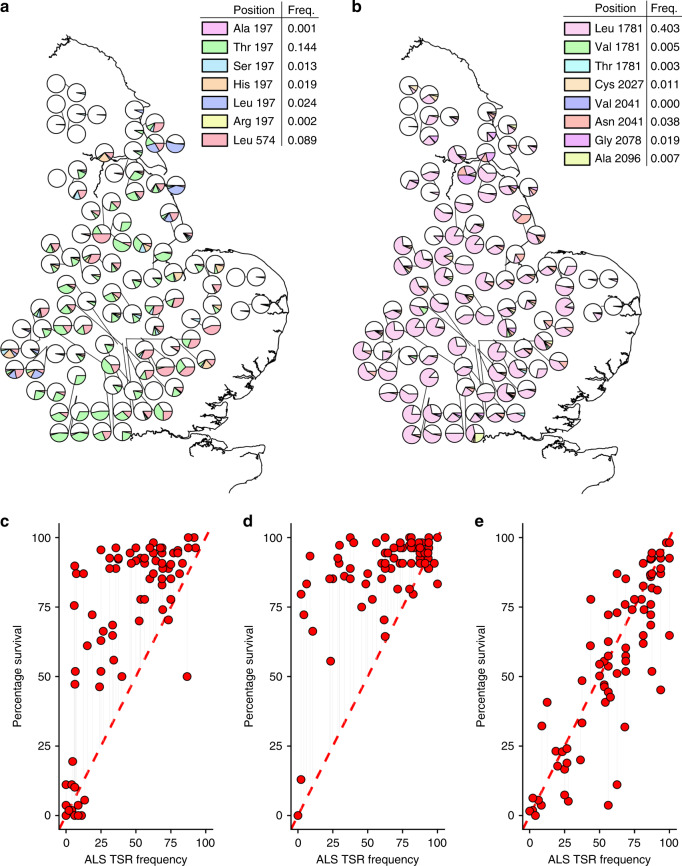
Contribution of ALS and ACCase target-site mutations to herbicide resistance across UK *A. myosuroides* populations. **a**, **b** Show the geographic distribution and relative frequency of amino acid substitutions in the ALS gene and ACCase gene, respectively. White portions of the pie charts show the frequency of wild-type sequence, while coloured portions represent the relative frequencies of each amino acid substitution. ‘Freq’ within the legend shows the overall allele frequency for each mutation across all tested populations. TSR genotype information was determined from ≥16 plants per population. Panels (**c**–**e**) show the observed proportion of individuals surviving herbicide (*Y*-axis) versus the proportion of individuals carrying a TSR resistance mutation (*X*-axis) for the ALS (SU), Fop and Dim herbicides, respectively. An expected 1:1 relationship is shown by the diagonal line. Points above the 1:1 line implicate the coexistence of TSR and NTSR mechanisms in respective populations. Source data are provided as a Source Data file.

Pyrosequencing (*n* = 2574 plants) identified multiple single nucleotide polymorphisms that are known to convey target-site (specialist) resistance to ACCase (Fop and Dim) and acetolactate synthase (ALS; SU) herbicides. Seven ALS resistance-endowing and eight ACCase resistance-endowing amino acid substitutions were identified, with mutations at position 197 of the ALS gene and 1781 of the ACCase gene being the most frequent (Fig. 2). Assuming that ACCase and ALS TSR mutations are dominant at field application rates^20^, we predicted the proportion of individuals in each population with a TSR phenotype for each herbicide (hereafter referred to as the ALS, Fop and Dim TSR frequencies). An approximate 1:1 relationship was observed between the Dim TSR frequency and observed survival at field dose of the herbicide cycloxydim (Fig. 2e), suggesting that this phenotype can be accounted for by specialist TSR alone. However, for the SU (Fig. 2c) and Fop (Fig. 2d) herbicides, observed survival was considerably higher than predicted from the TSR frequencies, suggesting the role of a further resistance mechanism in conferring the population-level resistance phenotype observed for these herbicides.

### Specialist and generalist mechanisms co-exist to confer resistance phenotypes

**Fig. 3:**
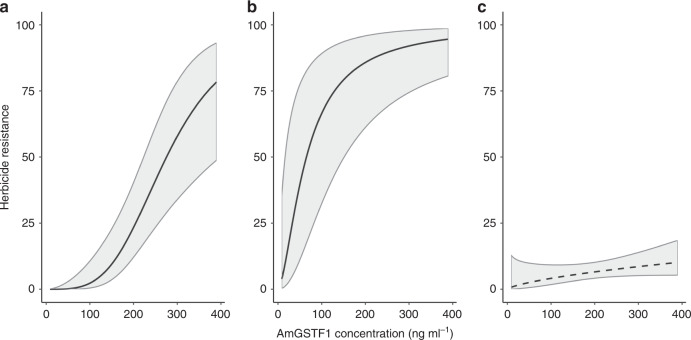
Modelled relationship between population-level phenotypic herbicide resistance and the protein biomarker AmGSTF1 for the generalist NTSR mechanism. *X*-axis shows the foliar concentration of the Am*GSTF1* protein, while *Y*-axis represents herbicide resistance as the proportional (%) survival of individuals for (**a**) the ‘SU’ herbicide mesosulfuron, (**b**) the ‘Fop’ fenoxaprop and (**c**) the ‘Dim’ cycloxydim. Fitted lines show the mean predicted relationship following mixed model analysis given in Supplementary Table 2, with shaded regions representing the estimated 95% confidence interval of the prediction. The modelled response variable was the proportional survival of individuals (*n* = 18) over all doses of the tested herbicide. Terms for the target-site resistance frequency, Am*GSTF1* concentration and their interaction were included as fixed factors, with each model containing random effect terms for the population ID and herbicide dose. Solid black lines are used for significant relationships in (**a**) (*p* ≤ 0.001) and (**b**) (*p* ≤ 0.001), while a dashed line is used where the relationship was non-significant in (**c**) (*p* = 0.10 ns).

‘Generalist’ NTSR in blackgrass is associated with the upregulation of herbicide-detoxifying enzymes, with increased expression of the glutathione-S-transferase Am*GSTF1* functionally linked to NTSR^32,45^. To characterise the NTSR status of all populations, mean population-level foliar concentrations of Am*GSTF1* were determined. A mixed model analysis identified that Am*GSTF1* content was a significant predictor of the population-level resistance phenotype for both fenoxaprop (Fop) and mesosulfuron (SU) resistance, but not for resistance to cycloxydim (Dim) (Supplementary Table 2, Fig. 3). These results provide further validation of this protein as a functional biomarker for NTSR in *A. myosuroides*. As expected, the mixed model confirmed that TSR frequencies were also highly significant as predictors of herbicide resistance (Supplementary Table 2). These results clearly establish that in populations sampled across England, specialist (TSR) and generalist (NTSR) mechanisms co-exist and account for observed herbicide resistance phenotypes to the SU and Fop herbicides.

### Herbicide mixtures preferentially selected for generalist resistance mechanisms

**Fig. 4:**
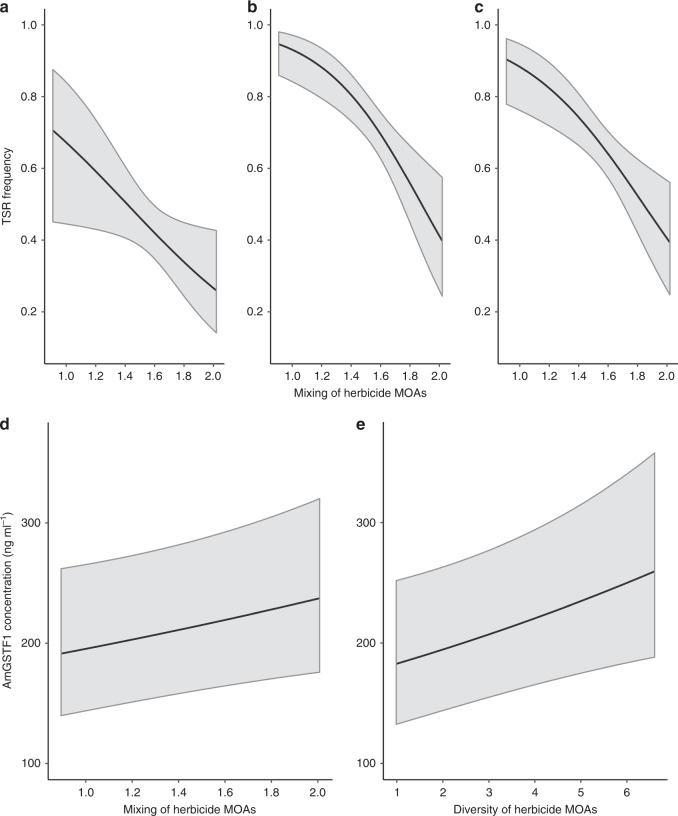
The effect of historical herbicide mixing and diversity on selection for target-site (TSR) and non-target-site resistance (NTSR) mechanisms. **a**–**c** show the mean predicted relationship between herbicide mixing and TSR frequency for the SU (*p* = 0.023), Fop (*p* ≤ 0.001) and Dim (*p* = 0.002) herbicides, respectively. Coefficients are from mixed model analyses fitted for each TSR separately (Supplementary Table 4). **d** The mean predicted relationship between the use of herbicide mode of action mixtures and the foliar concentration of Am*GSTF1* (*p* = 0.040) (used here as a proxy for NTSR), and **e** the mean predicted relationship between the overall diversity of herbicide modes of action and the foliar Am*GSTF1* concentration (*p* ≤ 0.001) following mixed model analysis are given in Supplementary Table 3. Solid lines show the mean predicted relationship, with shaded regions representing the 95% confidence interval of the prediction.

Herbicide diversity and the use of herbicide mixtures were significant positive predictors of the level of NTSR within populations, indicating that both practices preferentially selected for this generalist resistance mechanism (Fig. 4d, e, Supplementary Table 3). In contrast, a significant negative effect of herbicide mixing on the frequency of TSR was observed (Fig. 4a–c, Supplementary Tables 3 and 6). As predicted by theory, our results confirm that mixtures can constrain selection for specialist resistance. However, these results also reveal a potential trade-off in resistance management, whereby the use of mixtures to counter specialist, TSR, may impose a greater selective pressure for generalist, NTSR.

Our results are consistent, in that phenotypic resistance to ‘Dim’ herbicides is negatively associated with greater use of herbicide mixtures (Fig. 1f). This is because this ‘Dim’ resistance is caused solely by TSR mutations (Fig. 2e), and TSR mutations are selected against by herbicide mixtures (Fig. 4a–c). Conversely, phenotypic resistance to the ‘Fop’ and ‘SU’ herbicides showed no association with the use of herbicide mixtures (Fig. 1d, e). This is because resistance to these herbicides is conferred by a combination of ‘specialist’ TSR and ‘generalist’ NTSR mechanisms (Fig. 2c, d). In this case, the opposing effects of mixtures on TSR and NTSR (Fig. 4d, e), mean that there are no overall impacts of the use of herbicide mixtures on the resistance phenotype.

## Discussion

We have used an evolutionary epidemiological approach, based on national scale field and laboratory data, to demonstrate that ‘specialist’ target-site (TSR) and ‘generalist’ non-target-site (NTSR) herbicide resistance mechanisms co-exist in populations of blackgrass. Using this approach, we show clearly contrasting relationships between the historical intensity of the use of herbicide mixtures, and the presence of specialist, versus generalist, resistance mechanisms in *A. myosuroides*. When considered alongside emerging data from experimental evolution in other species, e.g.,^8,48,49^, these results support the theory that the use of pesticide mixtures may mediate an evolutionary trade-off between selection for specialist versus more generalist resistance mechanisms. Given the potential for generalist resistance mechanisms to evolve in other species and taxa, these results could have significant implications for the design of resistance management strategies in agriculture and healthcare.

Specifically, we found that the frequency of specialist TSR within both the ALS and ACCase genes was negatively associated with greater use of herbicide mixtures. Where TSR alone was the sole mechanism providing resistance (the ‘DIM’ herbicide), the population-level resistance phenotype was also negatively associated with greater use of herbicide mixtures, providing epidemiological support for previous empirical and theoretical studies that have established that heterogeneous selection environments can delay the evolution of resistance^37,50–53^. Where generalist adaptations can evolve, however, heterogeneous selection is predicted to promote the evolution of these traits^40,41^. In this study, we found that the elevated expression of Am*GSTF1*, a quantitative biomarker for certain types of NTSR in blackgrass^32,45^, was strongly correlated with resistance to both ACCase and ALS herbicides, confirming that this marker is associated with a generalist herbicide cross-resistance phenotype. Notably, we show that this resistance mechanism is positively associated with heterogeneous selection. Consequently for the ‘Fop’ and ‘SU’ herbicides, where both TSR and NTSR mechanisms contribute to resistance, we found no overall effect of the use of herbicide mixtures on the population-level resistance phenotype.

These results conform to ecological theories predicting the evolution of specialist and generalist adaptations^38–41^, and as such may be generalisable across taxa and chemical classes (pesticides, antimicrobials, anti-retrovirals and anti-cancer chemotherapy), wherever generalist resistance mechanisms can evolve. Whilst our data do not empirically prove a cause–effect relationship, there is mounting evidence from observational and experimental evolution studies in other species that this may be the case. In the bacterium *Pseudomonas aeruginosa*, combination therapy with β-lactam (ceftazidime) and fluoroquinolone (ciprofloxacin) antimicrobials was selected for broad spectrum (generalist) multidrug resistance, whereas single-drug exposure resulted in narrow-spectrum (specialist) resistance^48^. In another study of *P. aeruginosa*, combined antibiotic and phage therapy resulted in rapid selection for biofilm growth, providing a generalised resistance to both treatments^49^. In the unicellular algae *Chlamydomonas reinhardtii*, selection with a herbicide mixture resulted in a broader, more generalist cross-resistance than selection with the mixture components individually^8^. In addition, attempts to control the cockroach *Blattella germanica*, using insecticide mixtures, were ineffective, and led to the rapid evolution of a generalist cross-resistance mechanism^54^. Together, these studies demonstrate that where generalist mechanisms of resistance exist, these may be preferentially selected through the use of mixtures and MOA diversity.

The results of the current study demonstrate a potential resistance management trade-off: using mixtures to combat the evolution of specialist resistance may promote the evolution of a generalist resistance mechanism. In the current study, we have not attempted to compare the relative efficacy of different mixtures of specific actives. It is highly probable that there are combinations of chemical classes that are less prone to the evolution of generalist resistance, and where demonstrated, the use of pesticide and/or antibiotic mixtures should continue to be advocated as a rational resistance management strategy. Nevertheless, our study clearly demonstrates that effective resistance management is contingent on understanding the evolutionary potential for specialist versus generalist resistance traits, as these attributes may significantly alter the evolutionary response to much-promoted ‘best-management’ practices. In that respect, future control of pests, weeds and diseases will become increasingly reliant on rapid and accurate resistance diagnostics, in order to select the best combinations of chemical and non-chemical strategies to mitigate the evolution of resistance. Whilst pesticide (or antibiotic) diversity will remain a key strategy for managing the evolution of specialist resistance, the widespread occurrence of generalist resistance mechanisms questions the ubiquity of strategies based solely on MOA diversity, mixtures and/or combination therapy.

## Methods

### Seed collection and source populations

Blackgrass seeds were collected in 2014 from 132 wheat production fields from 71 farms across England^47^. Seeds were sampled using a stratified-random approach from ten locations within each field. At each location, seeds were collected from multiple plants, sampled from a circumference of ~5–10 m. A single representative seed population for each field was subsequently generated by combining 50% by weight of seed collected at all sampling locations within a field. These field-scale seed populations were used in all phenotypic and genotypic assays reported here. Blackgrass abundance in each field was visually recorded using a density-structured approach^55^, and with a density calculated per field on a scale of 0–4 (absent–very high).

### Glasshouse resistance phenotyping

Phenotypic herbicide resistance was established using glasshouse dose–response experiments conducted over October 2014–May 2015. Three herbicides were tested: a commercial formulation of mesosulfuron-methyl + iodosulfuron (site of action; ALS: chemical class; SU), fenoxaprop-p-ethyl (site of action; acetyl-CoA-carboxylase, ACCase: chemical class; aryloxyphenoxypropionate, ‘Fop’), and cycloxydim (site of action; acetyl-CoA-carboxylase, ACCase: chemical class; cyclohexanedione, ‘Dim’). All herbicides are registered for blackgrass control. For each herbicide, the dose–response design consisted of six treatments (five herbicide doses plus a no-herbicide control). For the ALS herbicide, ‘mesosulfuron’, doses were 2.7, 5.4, 10.8, 21.6 and 43.2 g a.i. ha^−1^ (current recommended UK field rate is 14.4 g a.i. ha^−1^), fenoxaprop doses were 17.19, 34.38, 68.75, 137.5 and 275 g a.i. ha^−1^ (current UK field rate is 68.75 g a.i. ha^−1^), while cycloxydim doses were 37.5, 75, 150, 300 and 600 g a.i. ha^−1^ (current UK field rate is 150 g a.i. ha^−1^).

Seeds from each population were pre-germinated in an incubator (Sanyo, MLR-350) with a 17/11 °C temperature cycle and a 14/10 h light/dark cycle, before sowing into 8 cm plastic plant pots filled with a Kettering loam soil, mixed with 2 kg m^−2^ osmocote fertiliser. Eighteen pots, each with six seedlings, were sown per population, providing three pots at each herbicide dose. Replicate pots were blocked over three glasshouse compartments, with the position of pots within each compartment determined using a randomised alpha design. The glasshouse was set to maintain ~16 °C during the day and 10 °C at night, with a 14/10 h day/night cycle. Three reference populations with known herbicide resistance phenotypes (herbicide susceptible, target-site resistant and non-target-site resistant) were included as positive and negative controls. Over the three dose–response experiments, we phenotyped 324 plants per population, with over 40,000 plants phenotyped in total. Plants were maintained in the glasshouse for 2–3 weeks until they reached the 3–4 leaf stage. At this point, pots were removed from the glasshouse and sprayed with herbicide using a fixed track sprayer with a Teejet 110015VK nozzle placed 50 cm above the plant canopy. The boom speed was set at 0.33 m s^−1^, and herbicide was applied at a volume of 197–213 L ha^−1^. After spraying, pots were immediately returned to the glasshouse. After 3 weeks, the number of surviving plants per pot was assessed, and above-ground tissue was harvested on a per pot basis. Leaf tissue was oven dried at 80 °C for 48 h before weighing to determine plant biomass.

### Target-site resistance sequencing

Known TSR mutations within the ALS and ACCase genes were analysed using pyrosequencing^56^. Approximately 2 cm of air-dried leaf material was homogenised in 400 µl of extraction buffer (100 mM Tris (HCl) and 1 M KCl at pH 9.5). A 5 µl aliquot of the resulting supernatant was diluted using 250 μl PCR grade water (Merck, Darmstadt, Germany) and used in PCR reactions containing HotStar Taq Master Mix (Qiagen, Hilden, Germany) and biotinylated primers. Two ALS fragments were amplified to include amino acid positions 197 or 574, sites at which resistance conferring mutations have previously been documented. Two ACCase fragments were amplified, the first of which included amino acid position 1781 and the second of which spanned positions 2027, 2041, 2078 and 2096; all sites where resistance conferring mutations have been reported. The biotinylated ALS and ACCase fragments were used in pyrosequencing reactions to amplify individual target-sites using site-specific primers. For pyrosequencing, 12 µl of amplified biotinylated PCR product was combined with a 70 µl solution containing binding buffer and streptavidin-coated Sepharose beads and shaken for 5 min, before washing and drying DNA coated beads at 80 °C. Sequencing was carried out using a Pyromark Q96 MD pyrosequencer (Qiagen, Hilden, Germany). In total, genotyping data were generated for 2574 individual plants (*n* ≥ 16 plants per population). All primer sequences, fragment lengths and PCR conditions are given in Supplementary Table 5. The geographical distribution of target-site mutations was visualised using ArcMaps (version 10).

To determine the importance of TSR mechanisms on the observed herbicide resistance phenotypes, we used the genotype sequencing (above) to calculate the proportion of individuals in each population which would be expected to survive each of the three tested herbicides, based on presence of TSR mutations alone. Whilst different ALS and ACCase mutations in *A. myosuroides* may vary in their protective efficacy, previously published information, e.g.,^20,57–59^ suggests that they can be considered dominant in conveying survival at field-relevant herbicide doses. Using this information, the proportion of individuals carrying TSR resistance to each herbicide was calculated, hereafter referred to as the ALS, Fop and Dim TSR frequencies. The ACCase 2096 Glycine—Alanine substitution is reported to convey variable levels of resistance to Dim herbicides^20^, but as some Dim resistance is reportedly provided by this mutation in *A. myosuroides* it was considered as a Dim resistance mutation in the current study.

### Quantification of AmGSTF1 protein abundance

To determine the importance of the ‘generalist’ NTSR for these herbicides, we quantified the foliar concentration of Am*GSTF1*; a phi-class glutathione-S-transferase protein which has been validated as a marker for NTSR in blackgrass^32,45^ (and see Supplementary Fig. 2). Seeds from each population were pre-germinated as previously described, and seedlings were sown into 8 cm plastic plant pots filled with a standard soil mix (see above). Three replicate pots were sown per population, each containing 15 seedlings, and were maintained within a single glasshouse compartment for 2 weeks until the plants reached the two-leaf stage. Glasshouse conditions were as described above, and the position of pots was fully randomised within the compartment. After 2 weeks of growth, the above-ground leaf and shoot material were harvested on a per replicate pot basis (*n* = 3) and immediately flash frozen in liquid nitrogen. Harvested tissue was stored at −80 °C until analysis.

Total protein was extracted from 100 mg of pulverised foliage tissue with 500 µL of cold protein extraction buffer (100 mM Tris-HCl, 150 mM NaCl and 5 mM EDTA, adjusted to pH 7.5 with NaOH, and with the addition by volume of 5% glycerol and 2% PVPP (polyvinylpolypyrrolidone)). The mixture was incubated on ice for 15 min, and centrifuged twice at 12,000 ×  *g*, 4 °C for 15 min. Total protein concentration was determined by Bradford assay (Bio-Rad protein assay kit, Bio-Rad, California). Protein concentration was calculated from a standard curve of bovine serum albumen (BSA).

Am*GSTF1* protein concentration was quantified by enzyme-linked immunosorbent assay using specific sheep antibodies for blackgrass GSTF1 protein. The 96-well microtiter plates were coated overnight at 4 °C with 100 µL of primary antibody (S909-D, diluted to 1 µg ml^−1^ in phosphate saline buffer). Plates were washed four times with phosphate saline buffer containing 0.1% tween 20 (PBS-T), and 200 µL PBS containing 1% BSA was added to each well to block unspecific binding of the antigen–antibody. Plates were incubated for 1 h at room temperature and then washed four times with PBS-T. 100 µl of plant protein samples was added and a dilution series of recombinant Am*GSTF1* protein (0–1000 ng mL^−1^) was also included on each plate to provide a standard curve for quantification. Plates were incubated for 1 h at room temperature in the vertical shaker (150 rpm). Plates were washed with PBS-T before addition of 100 µl of secondary antibody conjugated with horseradish peroxidase (S908D-HRP, diluted to 25 ng ml^−1^ in PBS-T). Plates were incubated in a vertical shaker for 1 h, then washed with PBS-T, before addition of 100 µl of a tetramethylbenzidine solution to each well. Plates were incubated in the dark at room temperature for 30 min. Absorbance at 655 nm was measured using a microplate reader (iMark, Bio-Rad). The reaction was stopped by addition of 50 µl of 1 M HCL and the absorbance at 450 nm was determined. Samples and standards were analysed in duplicate. The concentration of the Am*GSTF1* protein was calculated from standard curve (four-parametric logistic regression fitting) of recombinant Am*GSTF1* protein.

### Field management histories

Field management histories were collected for 94 of the 132 blackgrass populations^47^ and provide a mean of 7 years data on historical herbicide usage. These data were used to calculate indices of the intensity and heterogeneity of herbicide selection. To correspond with the herbicides used within the glasshouse phenotyping assays, measures of herbicide intensity were calculated as the mean annual number of applications of SU, ‘Fop’ and ‘Dim’ herbicides. Reflecting variance in both the number of year’s exposure, and the frequency of applications within year, herbicide intensity measures the degree to which each herbicide class has historically been used for each population. Herbicide diversity was measured as the mean number of different herbicide MOAs applied each year, giving an estimate of the range of herbicide MOAs used (see Supplementary Table 6). A measure of herbicide mixing was calculated as the mean number of different herbicide MOAs being applied on the same day. We made no distinction between mixtures applied at different times of year, or between pre-formulated herbicide products containing multiple MOAs versus separate single MOA herbicide products being applied simultaneously. Higher values indicate a greater likelihood that herbicides from any one MOA are applied alongside one or more herbicides from another MOA. In addition to these measures, the mean blackgrass density for each field was used as a measure of blackgrass population abundance, the proportion of years fields had been sown to autumn crops was calculated, and an index representing cultivation intensity was used, varying from 0 (no soil movement, e.g., direct drilling) to 4 (substantial soil movement, e.g., ploughing)^47^. All data were stored as comma separated value files before analysis (Microsoft Excel, version 15.0.5233.1000).

### Statistical analysis

A dose–response curve fitting approach was used for the herbicide phenotyping data (survival of *n* = 18 plants per dose, per population). Data from all field populations, overall three herbicide screening experiments were included, with the model fitting a separate curve for each herbicide. In this way, the fitted curve for each herbicide is estimated using data from all of the screened populations, providing a relative estimate of resistance to each herbicide at the national scale. Models were initially fitted with two-parameter log-logistic, Weibull type-1 and Weibull type-2 curve types. The two-parameter Weibull type-2 model was retained for analysis based on comparison of each models Akaike’s information criterion. For comparison, a dose–response model was also fitted for each herbicide using herbicide screening data from a fully susceptible standard population. To avoid problems with model convergence, three separate models were fitted for the standard susceptible population, one for each herbicide tested. Results from this fully herbicide susceptible population are provided for reference, to illustrate the extent of resistance evolution amongst the national collection of field blackgrass populations. All models were fitted using the ‘drc’ package^60^ in R version 3.4.2.

Generalised linear mixed models (GLMM) were used to analyse herbicide selective histories as predictors of herbicide resistance phenotype for the three herbicides. Survival of individuals at each dose (*n* = 18) was specified as the (binomial) response variable, with population ID and herbicide dose included as random factors. The measures of field selection history, including the calculated indices of herbicide intensity and heterogeneity, blackgrass abundance, and location were added sequentially as fixed effects to the models, and their significance estimated using parametric bootstrapping with the package ‘pbkrtest’ version 0.4–7^61^. In this way, the importance of herbicide mixtures is assessed from models containing fixed effect terms, which already account for variation in herbicide intensity and the overall diversity of herbicide products applied. Herbicide intensity (annual frequency of applications) was calculated separately for the SU, Fop and Dim herbicides, and in each case the corresponding herbicide intensity variable was used in models of SU, Fop or Dim resistance. Data for each herbicide were analysed separately, and models were fitted using the ‘lme4’ package^62^ in R version 3.4.2.

Further GLMM models were used to establish the significance of the TSR resistance frequency, Am*GSTF1* protein concentration, and their interaction, as predictors of phenotypic herbicide resistance (survival). Again, the survival data were modelled as a binomial response, with population ID and herbicide dose included as random factors. Terms representing the two resistance mechanisms and their interaction were added to models sequentially as fixed effects, and their significance estimated using parametric bootstrapping as above. The Am*GSTF1* variable was log-transformed before analysis.

A final set of mixed model analyses was used to assess the association between herbicide selective histories and the measures of TSR and NTSR resistance. For TSR, all three measures of TSR (the proportion of plants with TSR to the SU, Fop and Dim herbicides) were included as the response variable, and analysed using a binomial GLMM. A random effect term was included for the resistance MOA (SU, Fop and Dim), and a further random effect term for the population ID. For the ‘NTSR’ model, all three replicate measurements of the Am*GSTF1* protein concentration were included as the response variable, analysed using a linear mixed model. Am*GSTF1* protein concentrations were log-transformed before analysis. A random effect term was included for the ‘rep’, and a further random effect term for the population ID. In both models, as previously, field management variables representing the selective history were added sequentially as fixed effects, and their significance assessed using parametric bootstrapping with the package ‘pbkrtest’ version 0.4–7. The combined frequency of applications of SU, Fop and Dim herbicides was used as the measure of herbicide intensity. All models were fitted using the ‘lme4’ package^62^ in R version 3.4.2.

As a further test, the TSR models were then re-run for each TSR MOA (SU, Fop and Dim) separately, i.e., a separate model for TSR to the SU herbicides, the aryloxyphenoxypropionate herbicides (Fop) and the cyclohexanedione-oxime herbicides (Dim). In each case, model fitting and assessment of significance was as described above, with the exception that models were no longer fitted with a random effect term for the resistance MOA, as each MOA was analysed in a separate model. As in the models of phenotypic herbicide resistance, the annual frequency of applications of either SU, Fop or Dim herbicides was used as the measure of ‘herbicide intensity’ for the SU, Fop, or Dim TSR resistances, respectively. The overall results of all fitted mixed models are given within Supplementary Tables 1–4.

### Reporting summary

Further information on research design is available in the Nature Research Reporting Summary linked to this article.

## Supplementary information

Supplementary Information

Reporting Summary

## Data Availability

The data that support the findings of this study are available from the corresponding author upon reasonable request. The source data underlying Figs. 1–4 and Supplementary Figs. 1 and 2 are provided as a Source Data file.

## Source data

Source Data
