# Publisher Correction: Evolution of generalist resistance to herbicide mixtures reveals a trade-off in resistance management

**DOI:** 10.1038/s41467-020-18079-3

**Published:** 2020-09-02

**Authors:** David Comont, Claudia Lowe, Richard Hull, Laura Crook, Helen L. Hicks, Nawaporn Onkokesung, Roland Beffa, Dylan Z. Childs, Robert Edwards, Robert P. Freckleton, Paul Neve

**Affiliations:** 1 Department of Biointeractions and Crop Protection, Rothamsted Research, Harpenden, Hertfordshire, AL5 2JQ UK; 2 Department of Animal and Plant Sciences, University of Sheffield, South Yorkshire, S10 2TN UK; 3 School of Animal, Rural and Environmental Sciences, Nottingham Trent University, Southwell, NG25 0QF UK; 4 School of Natural and Environmental Sciences, Newcastle University, Newcastle, NE1 7RU UK; 5 Bayer Crop Science, Weed Resistance Research, 65926 Frankfurt, Germany; 6 Agriculture & Horticulture Development Board, Stoneleigh Park, Kenilworth, CV8 2TL UK

**Keywords:** Agroecology, Evolutionary theory, Plant evolution

Correction to: *Nature Communications* 10.1038/s41467-020-16896-0, published online 18 June 2020.

The original version of this Article contained errors in Fig. 2d, e, in which the *x*-axis labels were incorrect.

The correct version of Fig. 2:

**Figure 1:**
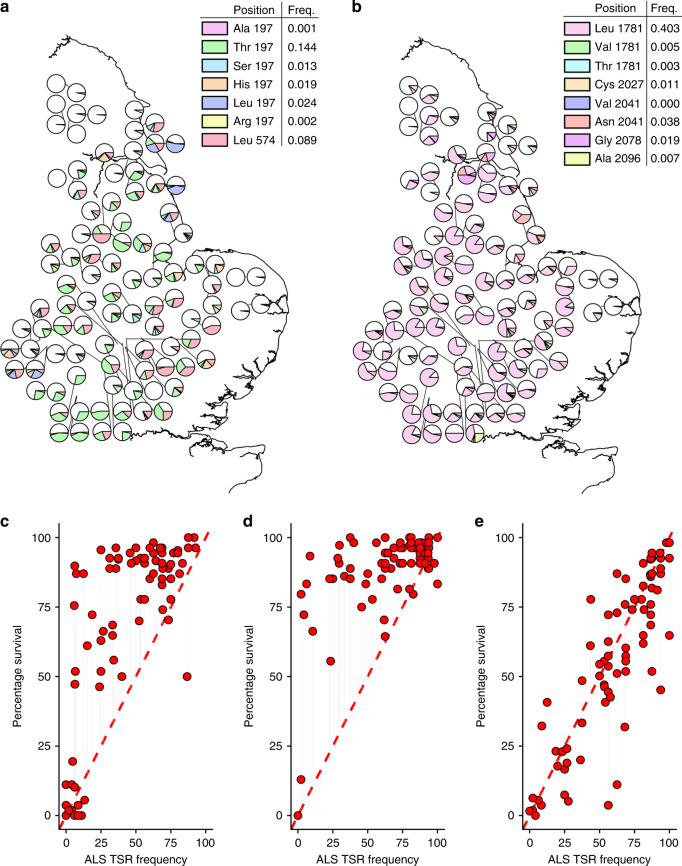
Fig. 2

which replaces the previous incorrect version.

**Figure 2:**
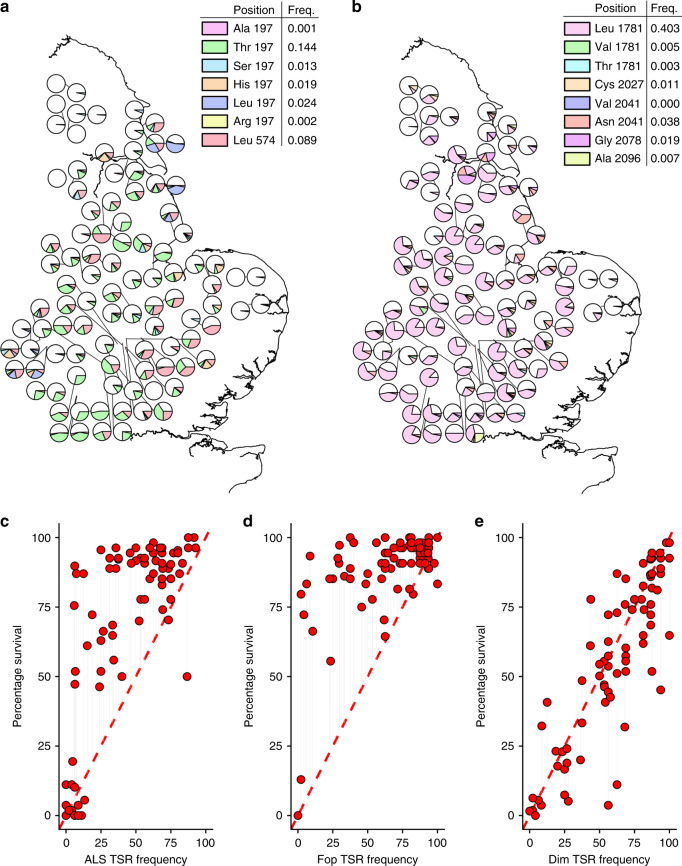
Fig. 2

This has been corrected in both the PDF and HTML versions of the Article.

