# Supplementary material for: Evolution of generalist resistance to herbicide mixtures reveals a trade-off in resistance management
Source: Nat Commun. 2020 Jun 18;11:3086. doi: 10.1038/s41467-020-16896-0 (PMC7303185; doi:10.1038/s41467-020-16896-0)
Supplement: Supplementary file 1 — Supplementary Information [file 41467_2020_16896_MOESM1_ESM.pdf]

Supplementary Information for

Evolution of generalist resistance to herbicide mixtures reveals a trade-off in resistance management

Comont et al.

This file includes:

Supplementary Figs. 1 to 2

Supplementary Tables 1 to 6

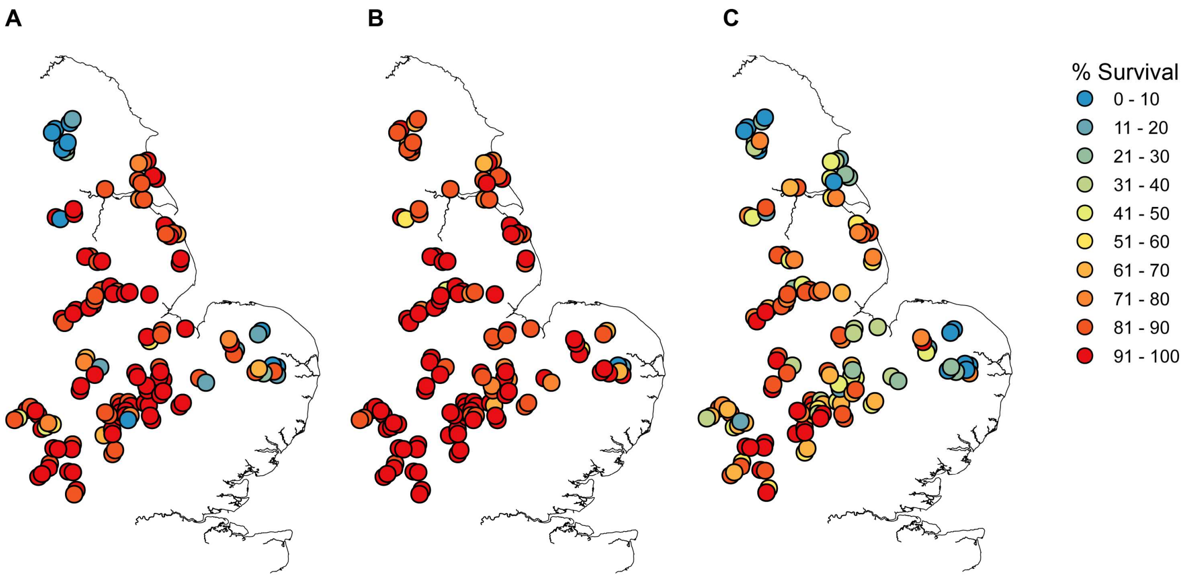

Supplementary Fig. 1: Maps showing the frequency and geographical distribution of resistance for (**A**) mesosulfuron, (**B**) fenoxaprop, and (**C**) cycloxydim. In each case, percentage survival was calculated from the dose closest to field rate. Source data are provided as a Source Data file.

Supplementary Table 1: Binomial generalized linear mixed-model analysis of the effect of field management and population variables on herbicide resistance in UK *A. myosuroides* populations. The response variable was the proportional survival of individuals (n=18) across five doses of the herbicides mesosulfuron, fenoxaprop, and cycloxydim. Field management history variables were included as fixed factors, with each model containing random effect terms for the population identity and herbicide dose. 'Herbicide intensity' represents the annual frequency of applications of either SU, Fop or Dim herbicides as predictors of the SU, Fop, or Dim resistance, respectively. Herbicide diversity and herbicide mixing represent the mean number of different herbicide modes-of-action applied in a single year (diversity) or on the same day within a given year (mixing), with higher values signifying greater heterogeneity of selection. Individual field management variables were included sequentially into the model, with P-values calculated using parametric bootstrapping (with the package 'pbkrtest' [64]).

	**'SU' resistance** (survival of mesosulfuron)				**'Fop' resistance** (survival of fenoxaprop)				**'Dim' resistance** (survival of cycloxydim)			
**Predictor**	**Est.**	**SE**	**SSq**	**P-val**	**Est.**	**SE**	**SSq**	**P-val**	**Est.**	**SE**	**SSq**	**P-val**
Herbicide usage												
Herbicide Intensity	0.26	0.205	12.3	0.006 **	0.26	0.104	7.00	0.040 *	0.29	0.121	25.9	0.001 **
Herbicide Diversity	0.23	0.211	3.25	0.142	0.09	0.106	2.28	0.211	0.21	0.132	2.83	0.187
Herbicide Mixing	-0.07	0.188	1.64	0.256	-0.00	0.093	1.50	0.265	-0.47	0.124	21.8	0.001 **
Cultivation												
Cultivation depth	-0.02	0.185	2.38	0.223	0.03	0.094	1.88	0.274	-0.09	0.121	4.51	0.099
Prop. Autumn sown	0.01	0.187	1.71	0.300	-0.11	0.099	7.63	0.023 *	-0.01	0.124	3.13	0.150
Population												
Blackgrass abundance	1.02	0.183	33.8	0.002 **	0.19	0.095	6.18	0.042 *	0.30	0.119	9.56	0.014 *
Latitude	-0.39	0.191	4.76	0.056	-0.41	0.098	18.1	0.001 **	-0.55	0.131	19.3	0.001 **
Longitude	-0.30	0.180	2.85	0.132	-0.26	0.093	7.47	0.020 *	-0.21	0.121	2.92	0.130

Supplementary Table 2: Binomial generalized linear mixed model analysis of population-level target-site resistance (TSR) and non-target-site resistance (NTSR, as determined by Am*GSTf1* concentration) mechanisms as predictors of the herbicide resistance phenotype. The herbicide resistance variable was the proportional suvival of individuals (n=18) over all doses of the herbicides mesosulfuron, fenoxaprop, and cycloxydim. Terms for the two resistance mechanisms and their interaction were included as fixed factors, with each model containing random effect terms for the population identity and herbicide dose. The significance of terms was tested sequentially, with P-values calculated using parametric bootstrapping (with the package 'pbkrtest' [64])

Herbicide	Predictor	Est	SE	Sum Sq	P-value
SU (mesosulfuron)	TSR freq	20.781	6.322	91.211	0.001 **
	AmGSTf1	20.130	5.123	6.266	0.001 **
	TSR x GSTf1	-17.926	6.297	7.852	0.005 **
FOP (fenoxaprop)	TSR freq	9.189	2.857	50.047	0.001 **
	AmGSTf1	8.683	1.998	11.903	0.001 **
	TSR x GSTf1	-7.597	2.867	6.971	0.013 *
DIM (cycloxydim)	TSR freq	5.245	2.957	222.278	0.001 **
	AmGSTf1	3.87	2.522	2.403	0.106
	TSR x GSTf1	-1.925	2.95	0.417	0.561

Supplementary Table 3: Mixed-model analysis of the effect of field management and population variables on the levels of TSR and NTSR mechanisms in blackgrass populations. The foliar Am*GSTf1* protein concentration was used as a proxy for NTSR, while the proportion of individuals carrying a TSR mutation to the ALS, Fop, and Dim herbicides was used as measures of TSR. The TSR model was fitted using a binomial generalized linear mixed model, while the NTSR model was fitted as a linear mixed model. Field management history variables were included as fixed factors, and population identity as a random factor. The NTSR model contained an additional random factor reflecting the three replicate Am*GSTf1* measurements taken per population, while the TSR model contained a random term for the resistance mode-of-action (ALS, FOP, DIM). The significance of individual terms was assessed using sequential fitting, with P-values calculated using parametric bootstrapping (with the package 'pbkrtest' [64])

	**TSR** (freq. of plants with TSR mutations)				**NTSR** (Am*GSTF1* protein concentration)			
**Predictor**	**Est**	**SE**	**SSq**	**P-val**	**Est**	**SE**	**SSq**	**P-val**
Herbicide usage								
Herbicide Intensity	0.425	0.090	27.2	0.001 **	-0.052	0.062	0.49	0.421
Herbicide Diversity	0.338	0.127	4.10	0.122	0.155	0.064	9.59	0.001 **
Herbicide Mixing	-0.495	0.125	20.7	0.001 **	0.114	0.056	2.64	0.040 *
Cultivation								
Cultivation depth	0.015	0.119	1.59	0.304	0.026	0.054	0.00	0.939
Autumn sown	-0.083	0.125	3.37	0.135	0.026	0.056	0.02	0.848
Population								
Blackgrass abundance	0.287	0.117	8.41	0.019 *	0.067	0.052	1.50	0.142
Latitude	-0.358	0.128	9.74	0.012 *	-0.074	0.057	1.21	0.163
Longitude	-0.224	0.123	3.35	0.128	-0.022	0.054	0.11	0.695

Supplementary Table 4: Binomial generalized linear mixed-model analysis of the effect of field management and population variables on separate estimates of target-site-resistance (TSR) to the ALS, Fop, and Dim herbicides. Field management history variables were included as fixed factors, and population identity as a random factor. The significance of individual terms was assessed using sequential fitting, with P-values calculated using parametric bootstrapping (with the package 'pbkrtest' [64])

	'SU' TSR				'Fop' TSR				'Dim' TSR			
**Predictor**	**Est.**	**SE**	**SSq**	**P-val**	**Est.**	**SE**	**SSq**	**P-val**	**Est.**	**SE**	**SSq**	**P-val**
Herbicide usage												
Herbicide Intensity	0.17	0.184	5.037	0.072	-0.16	0.190	0.150	0.816	0.35	0.154	17.86	0.001 **
Herbicide Diversity	0.46	0.191	3.654	0.109	0.48	0.182	3.409	0.132	0.31	0.163	1.083	0.390
Herbicide Mixing	-0.39	0.176	6.441	0.023 *	-0.66	0.170	17.34	0.001 **	-0.54	0.159	13.59	0.002 **
Cultivation												
Cultivation depth	0.13	0.163	0.032	0.859	-0.01	0.161	1.343	0.329	-0.04	0.150	2.200	0.207
Prop. Autumn sown	-0.25	0.174	3.871	0.088	-0.01	0.170	1.481	0.297	0.01	0.157	0.894	0.382
Population												
Blackgrass abundance	0.58	0.162	13.74	0.003 **	0.15	0.161	2.158	0.204	0.23	0.148	3.536	0.094
Latitude	-0.18	0.178	1.587	0.269	-0.52	0.174	10.24	0.007 **	-0.45	0.165	9.202	0.010 *
Longitude	-0.22	0.172	1.634	0.249	-0.15	0.166	0.847	0.425	-0.27	0.154	3.135	0.135

Supplementary Table 5: Pyrosequencing conditions and primer sequences for analysis of herbicide target-site mutations.

**Primer sequences**		Fragment amplification		Mutation detection
Gene	codon	Forward Primer	Reverse primer	Sequencing Primer
ALS	197	5’-GTGCTACCAACCTCGTCTC-3’	5’-GGAGCGGGTGACCTCTACAAT-3’	5’-ATGGTCGCTATCACGGGACAGGTT-3’
	574	5’-GCACACAAGATGCAGCTAGATAGT-3’	5’- TCCGATTCCAACAGTTCGT-3’	5’-CAACATCTGGGAATGGTGGTGCAG-3’
ACCase	1781	5’-GCACACAAGATGCAGCTAGATAGT-3’	5’- TCCGATTCCAACAGTTCGT-3’	5’-ATGGACTAGGTGTGGAGAAC-3’
	2027	5’-TCCTGTTGGTGTTATAGCTG-3’	5’-GGATCAAGCCTACCCATGCA-3’	5’-CCTCTGTTCATACTTGCTAAC-3’
	2041			5’-GCAAAGAGATCTTTTTGAAGGA-3’
	2078			5’-GTGGAGGAGCCTGGGTCGTGATT-3’
	2096			5’-GCTATGCTGAGAGGACTGCAAAG-3’

**PCR conditions**						
Gene	codon	Fragment length (bp)	Annealing	Denaturing	Elongation	No Cycles
ALS	197	140	94°C (30 sec)	60°C (30 sec)	72°C (40 sec)	45
	574	401	94°C (30 sec)	54.2°C (30 sec)	72°C (40 sec)	45
ACCase	1781	182	94°C (30 sec)	54°C (30 sec)	72°C (40 sec)	45
	2027, 2041, 2078, 2096	481	94°C (30 sec)	54.1°C (30 sec)	72°C (40 sec)	45

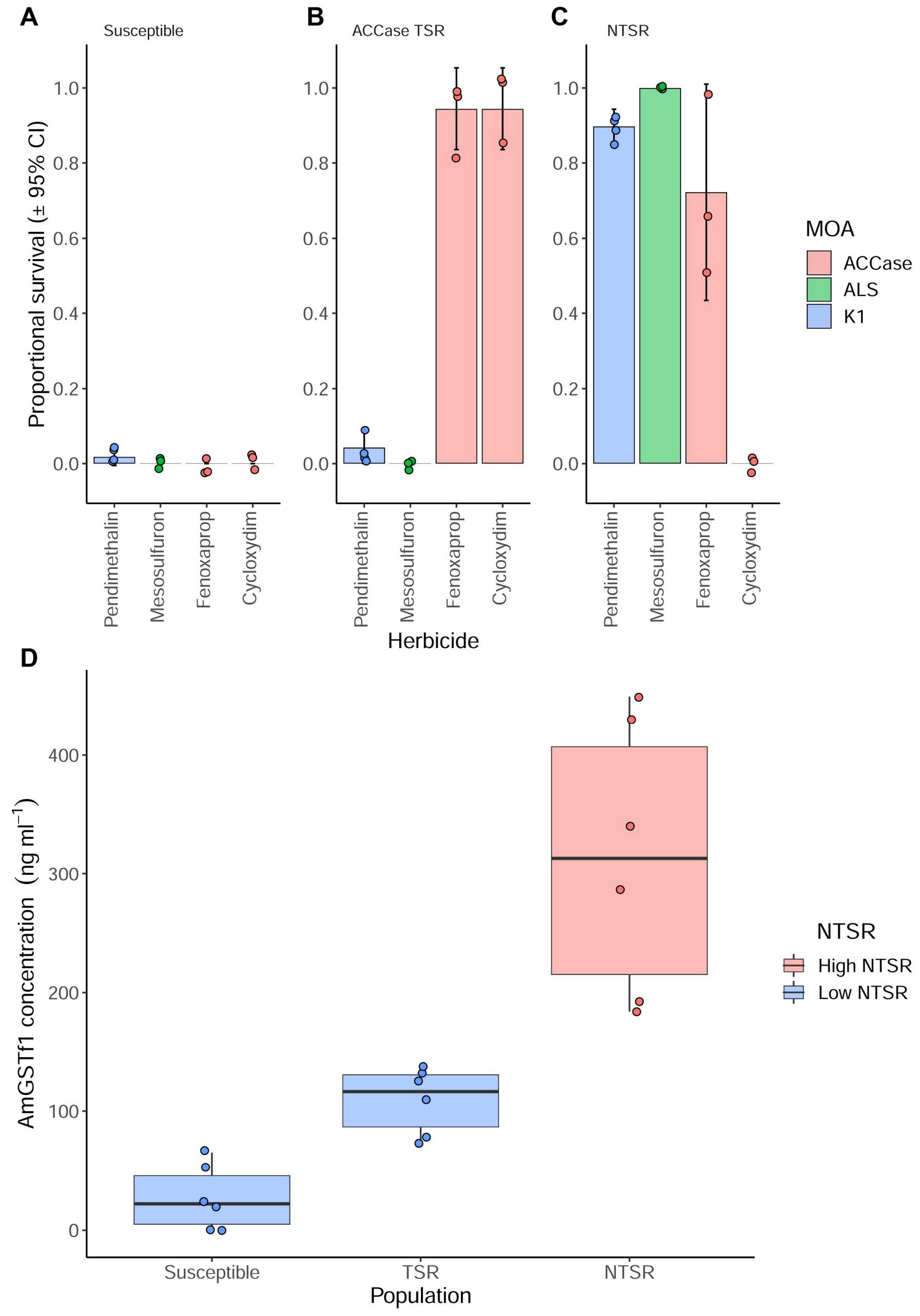

Supplementary Fig. 2: The association between specialist (TSR) and generalist (NTSR) resistance mechanisms and AmGSTf1 protein concentration in *A. myosuroides*. Cross resistance, measured as percentage survival (n=3 biologically independent samples) to the herbicides pendimethalin, mesosulfuron, fenoxaprop, and cycloxydim are shown for (**A**) a known susceptible population, (**B**) a population with ACCase target-site resistance, and (**C**) a population with non-target-site resistance. Error bars show the 95% onfidence interval about the mean. (**D**) shows the foliar concentration of the protein AmGSTf1 in these populations (n=6 biologically independent samples). The centre of each box shows the median, box minima and maxima represent the 25% and 75% quartiles, while whiskers depict the range of values. All assays were conducted once on seedlings grown in pots under glasshouse conditions (mesosulfuron, fenoxaprop, cycloxydim) or Petri-dishes (pendimethalin), and show the potentially broader cross-resistance profile and higher AmGSTf1 protein concentration associated with NTSR. Source data are provided as a Source Data file.

Supplementary Table 6: Herbicidal active ingredients found within the field management data, and associated mode of action and chemical class as defined by the Herbicide Resistance Action Committee (HRAC).

**Active**	**HRAC**	**Active group**	**Active type**
clethodim	A	ACCase inhibitors	Cyclohexanediones
clodinafop-propargyl	A	ACCase inhibitors	Aryloxyphenoxypropionates
cycloxydim	A	ACCase inhibitors	Cyclohexanediones
diclofop-methyl	A	ACCase inhibitors	Aryloxyphenoxypropionates
fenoxaprop-P-ethyl	A	ACCase inhibitors	Aryloxyphenoxypropionates
fluazifop-P-butyl	A	ACCase inhibitors	Aryloxyphenoxypropionates
pinoxaden	A	ACCase inhibitors	Phenylpyrazoline
propaquizafop	A	ACCase inhibitors	Aryloxyphenoxypropionates
quizalofop-P-ethyl	A	ACCase inhibitors	Aryloxyphenoxypropionates
tepraloxydim	A	ACCase inhibitors	Cyclohexanediones
tralkoxydim	A	ACCase inhibitors	Cyclohexanediones
amidosulfuron	B	ALS inhibitors	Sulfonylureas
florasulam	B	ALS inhibitors	Triazolopyrimidines
flupyrsulfuron-methyl-sodium	B	ALS inhibitors	Sulfonylureas
imazamox	B	ALS inhibitors	Imidazolinones
iodosulfuron-methyl-sodium	B	ALS inhibitors	Sulfonylureas
mesosulfuron-methyl	B	ALS inhibitors	Sulfonylureas
metsulfuron-methyl	B	ALS inhibitors	Sulfonylureas
nicosulfuron	B	ALS inhibitors	Sulfonylureas
propoxycarbazone-sodium	B	ALS inhibitors	Sulfonylaminocarbonyltriazolinone
pyroxsulam	B	ALS inhibitors	Triazolopyrimidines
rimsulfuron	B	ALS inhibitors	Sulfonylureas
sulfosulfuron	B	ALS inhibitors	Sulfonylureas
thifensulfuron-methyl	B	ALS inhibitors	Sulfonylureas
tribenuron-methyl	B	ALS inhibitors	Sulfonylureas
triflusulfuron-methyl	B	ALS inhibitors	Sulfonylureas
chloridazon	C1	Photosystem II inhibitors	Pyridazinones
cyanazine	C1	Photosystem II inhibitors	Triazines
desmedipham	C1	Photosystem II inhibitors	Phenyl-carbamates
lenacil	C1	Photosystem II inhibitors	Uracils
metamitron	C1	Photosystem II inhibitors	Triazinones
metribuzin	C1	Photosystem II inhibitors	Triazinones
phenmedipham	C1	Photosystem II inhibitors	Phenyl-carbamates
simazine	C1	Photosystem II inhibitors	Triazines
terbuthylazine	C1	Photosystem II inhibitors	Triazines
terbutryn	C1	Photosystem II inhibitors	Triazines
chlorotoluron	C2	PSII inhibitor	Ureas
isoproturon	C2	PSII inhibitor	Ureas
linuron	C2	PSII inhibitor	Ureas
bentazone	C3	PSII inhibitors	Benzothiadiazinones
bromoxynil	C3	PSII inhibitors	Nitriles

ioxynil	C3	PSII inhibitors	Nitriles
pyridate	C3	PSII inhibitors	Phenyl-pyridazines
diquat	D	PSI Electron Diverter	Bipyridyliums
bifenox	E	PPO inhibitors	Diphenylethers
carfentrazone-ethyl	E	PPO inhibitors	Triazolinones
flumioxazin	E	PPO inhibitors	N-phenylphthalimides
pyraflufen-ethyl	E	PPO inhibitors	Phenylpyrazoles
diflufenican	F1	Carotenoid biosynthesis inhibitors	Pyridinecarboxamides
flurtamone	F1	Carotenoid biosynthesis inhibitors	Others
picolinafen	F1	Carotenoid biosynthesis inhibitors	Pyridinecarboxamides
mesotrione	F2	HPPD inhibitors	Callistemones
clomazone	F4	DOXP inhibitors	Isoxazolidinones
glyphosate	G	EPSP synthase inhibitors	Glycines
glufosinate-ammonium	H	Glutamine synthase inhibitors	Phosphinic acids
pendimethalin	K1	Microtubule inhibitors	Dinitroanilines
propyzamide	K1	Microtubule inhibitors	Benzamides
trifluralin	K1	Microtubule inhibitors	Dinitroanilines
carbetamide	K2	Mitosis inhibitors	Carbamates
dimethenamid-p	K3	Long chain fatty acid inhibitors	Chloroacetamides
flufenacet	K3	Long chain fatty acid inhibitors	Oxyacetamides
metazachlor	K3	Long chain fatty acid inhibitors	Chloroacetamides
napropamide	K3	Long chain fatty acid inhibitors	Acetamides
isoxaben	L	Cellulose inhibitors	Benzamides
ethofumesate	N	Lipid Inhibitors (thiocarbamates)	Benzofuranes
prosulfocarb	N	Lipid Inhibitors (thiocarbamates)	Thiocarbamates
triallate	N	Lipid Inhibitors (thiocarbamates)	Thiocarbamates
clopyralid	O	Synthetic Auxins	Pyridine carboxylic acids
dicamba	O	Synthetic Auxins	Benzoic acids
fluroxypyr	O	Synthetic Auxins	Pyridine carboxylic acids
MCPA	O	Synthetic Auxins	Phenoxy-carboxylic-acids
MCPB	O	Synthetic Auxins	Phenoxy-carboxylic-acids
mecoprop-P	O	Synthetic Auxins	Phenoxy-carboxylic-acids
picloram	O	Synthetic Auxins	Pyridine carboxylic acids
quinmerac	O	Synthetic Auxins	Quinoline carboxylic acids
