# Supplementary material for: Evolution of generalist resistance to herbicide mixtures reveals a trade-off in resistance management
Source: Nat Commun. 2020 Jun 18;11:3086. doi: 10.1038/s41467-020-16896-0 (PMC7303185; doi:10.1038/s41467-020-16896-0)
Supplement: Supplementary file 2 — Reporting Summary [file 41467_2020_16896_MOESM2_ESM.pdf]

nature research

Corresponding author(s): David Comont
Last updated by author(s): May 13, 2020

Reporting Summary

Nature Research wishes to improve the reproducibility of the work that we publish. This form provides structure for consistency and transparency in reporting. For further information on Nature Research policies, see Authors & Referees and the Editorial Policy Checklist .

Statistics

For all statistical analyses, confirm that the following items are present in the figure legend, table legend, main text, or Methods section.

n/a	Confirmed	
☐	☒	The exact sample size (*n*) for each experimental group/condition, given as a discrete number and unit of measurement
☐	☒	A statement on whether measurements were taken from distinct samples or whether the same sample was measured repeatedly
☐	☒	The statistical test(s) used AND whether they are one- or two-sided<br>*Only common tests should be described solely by name; describe more complex techniques in the Methods section.*
☐	☒	A description of all covariates tested
☐	☒	A description of any assumptions or corrections, such as tests of normality and adjustment for multiple comparisons
☐	☒	A full description of the statistical parameters including central tendency (e.g. means) or other basic estimates (e.g. regression coefficient) AND variation (e.g. standard deviation) or associated estimates of uncertainty (e.g. confidence intervals)
☐	☒	For null hypothesis testing, the test statistic (e.g. *F*, *t*, *r*) with confidence intervals, effect sizes, degrees of freedom and *P* value noted<br>*Give P values as exact values whenever suitable.*
☒	☐	For Bayesian analysis, information on the choice of priors and Markov chain Monte Carlo settings
☐	☒	For hierarchical and complex designs, identification of the appropriate level for tests and full reporting of outcomes
☒	☐	Estimates of effect sizes (e.g. Cohen's *d*, Pearson's *r*), indicating how they were calculated

Our web collection on statistics for biologists contains articles on many of the points above.

Software and code

Policy information about availability of computer code

Data collection	Pyromark Q96 ID software for SNP identification. Microsoft Excel (version 15.0.5233.1000) for storing data
Data analysis	R version 3.4.2, package ‘lme4’ version 1.1-14, package ‘drc’ version 3.0-1, package ‘pbkrtest’ version 0.4-7, ArcMap version 10

For manuscripts utilizing custom algorithms or software that are central to the research but not yet described in published literature, software must be made available to editors/reviewers. We strongly encourage code deposition in a community repository (e.g. GitHub). See the Nature Research guidelines for submitting code & software for further information.

Data

Policy information about availability of data

All manuscripts must include a data availability statement. This statement should provide the following information, where applicable:

- Accession codes, unique identifiers, or web links for publicly available datasets
- A list of figures that have associated raw data
- A description of any restrictions on data availability

The data that support the findings of this study are available from the corresponding author upon reasonable request. The source data underlying Figs 1-4 and Supplementary Figs 1-2 are provided as a Source Data file.

Field-specific reporting

Please select the one below that is the best fit for your research. If you are not sure, read the appropriate sections before making your selection.

☐ Life sciences ☐ Behavioural & social sciences ☒ Ecological, evolutionary & environmental sciences

Ecological, evolutionary & environmental sciences study design

All studies must disclose on these points even when the disclosure is negative.

Study description	The study is an epidemiological assessment of the relationship between in-field weed management practices and the extent and mechanism of herbicide resistance. Based on assessment of 132 field populations of blackgrass, 94 of which have field history data. Resistance was assessed via assays of herbicide survival (18 plants per dose over five herbicide doses for each population). Target-site mechanisms were assessed using standard genotyping of ALS and ACCase genes (≥ 16 plants per population). NTSR was assessed from AmGSTf1 protein concentration (3 reps of 15 plants per population).
Research sample	The organism studied was Blackgrass (Alopecurus myosuroides), collected as seeds from UK agricultural fields. The samples used were collected from a previously established network of UK blackgrass field populations.
Sampling strategy	The sample sizes for the survey work were based on previous epidemiological analysis of similar size and scope (see Queenborough et al. 2011, Methods in Ecology and Evolution. 2, 289–302, and Hicks et al. 2018. Nature Ecology and Evolution. 2, 529–536). Seed sampling was conducted using a stratified-random approach from ten locations within each field. The sample size for the lab and glasshouse experiments were based on previous experiments in this species which have yielded reliable results (see Comont et al. New Phytologist. 223(3), 1584–1594). For herbicide experiments, replicate pots were blocked over three glasshouse compartments, with the position of pots within each compartment determined using a randomised alpha design. Samples for protein analysis were immediately flash-frozen in liquid N and stored at -80C. Samples for pyrosequencing were air-dried before analysis
Data collection	D.Comont and H.Hicks led the field monitoring and collection of seed populations and farm management data. D.Comont, L.Crook, and R.Hull performed the glasshouse experiments. C.Lowe performed the pyrosequencing. D.Comont performed protein extractions, and N.Onkokesung performed the quantification of AmGSTF1 protein concentration. Blackgrass abundance was recorded in contiguous 20x20m quadrats across each field, while seeds were collected from multiple plants across 10 locations per field, sampled from a circumference of approximately 5-10m. Herbicide spraying was performed using a custom-built track sprayer with a Teejet 110015VK nozzle. Tissue for protein and pyrosequencing analysis was collected by excising leaf material using scissors. Pyrosequencing results were taken using a Pyromark Q96 MD pyrosequencer, while protein concentration was determined using a microplate reader (iMark, BioRad).
Timing and spatial scale	Analyses are based on a network of 132 field populations of blackgrass, distributed across Eastern England from Hertfordshire in the South to Yorkshire in the North. Seeds were collected at a single pre-harvest time-point from each field, between July - early August 2014. Collection at this time ensured that only mature seed heads were sampled. All subsequent analyses were performed on plants grown from these seed populations. Glasshouse assays were timed to coincide with times of blackgrass vegetative growth in-field. In particular, herbicide assays were conducted over October 2014 – May 2015. Undertaking experimentation at this time ensures that ambient temperatures and light levels can be controlled more appropriately. Experimental durations were based on those determined from previous experimentation in this species (see e.g. Davies and Neve 2017, Weed research 57: 323-332, and Comont et al. New Phytologist. 223(3), 1584–1594), and represented 6-7 days pre-germination, 2-3 weeks growth to the three-leaf stage, and 3 weeks post spraying.
Data exclusions	Data were excluded from farms from which we were unable to obtain appropriate field management data. We made repeated attempts to obtain all such data.
Reproducibility	This was predominantly an observational study based on epidemiological associations between field populations and management histories. Individual experiments were not repeated, however the combined phenotypic resistance data is based on three separate glasshouse assays testing a different herbicide each time, with over 40,000 plants phenotyped in total. In all cases, appropriate positive and negative controls were used, such as using standard populations of known phenotype to validate results, and appropriate replication was used across all experiments. All population-level phenotypes were assessed from multiple, replicated individuals, for example with >100 individuals screened per population per herbicide.
Randomization	Seeds from across a single field were designated as a single population. Position of plants grown from these seed populations in the glasshouse was randomised using an alpha design
Blinding	Observational study: we did not assign to groups. Each population was assigned a unique but uninformative numerical code throughout analysis

Did the study involve field work? ☒ Yes ☐ No

Field work, collection and transport

Field conditions	Seeds were collected from Winter wheat fields pre-harvest in summer 2014 at the time of Blackgrass seed maturity and shedding (July - early August)
Location	Fields spanned a range of locations over Eastern England, from Oxfordshire/Hertfordshire in the South, to Yorkshire in the North
Access and import/export	Permission was sought and granted from individual land owners before accessing any private land, and before the collection and analysis of seed populations.
Disturbance	All care was taken to avoid any damage to the crop.

Reporting for specific materials, systems and methods

We require information from authors about some types of materials, experimental systems and methods used in many studies. Here, indicate whether each material, system or method listed is relevant to your study. If you are not sure if a list item applies to your research, read the appropriate section before selecting a response.

Materials & experimental systems

n/a	Involved in the study
☐	☒ Antibodies
☒	☐ Eukaryotic cell lines
☒	☐ Palaeontology
☒	☐ Animals and other organisms
☒	☐ Human research participants
☒	☐ Clinical data

Methods

n/a	Involved in the study
☒	☐ ChIP-seq
☒	☐ Flow cytometry
☒	☐ MRI-based neuroimaging

Antibodies

Antibodies used

AmGSTF1 protein concentration was quantified by Enzyme-Linked Immunosorbent Assay (ELISA) using specific sheep-antibodies for black-grass GSTF1 protein. The primary AmGSTF1 antibody (batch name S909D) and secondary AmGSTF1 antibody conjugated with horseradish peroxidase (batch name S908D-HRP) were stored in PBS buffer pH 7.4 at 4C. The primary and secondary AmGSTF1 antibodies were manufactured by Mologic Ltd. These two antibodies are customized antibodies and produced specifically for the project; hence they are not commercially available. The primary AmGSTF1 antibody (S909D- stock 1.1 mg/mL) is diluted in PBS to the final concentration of 1ug/mL and 100uL of diluted primary antibody is used per well. The secondary AmGSTF1 antibody (S908D-HRP stock 500 ug/mL) is diluted in PBS-T (0.1% Tween 20 v/v) to the final concentration of 25ng/mL and 100uL of the diluted secondary antibody is used per well.

Validation

AmGSTF1 antibodies were validated by Mologic Ltd using peptide array and titration to identify the suitable antibody pair (captor-detection) and the concentration of antibodies used for ELISA assay to detect AmGSTF1 from total plant protein samples. Additionally, we independently validated AmGSTF1 antibodies specificity using immunoblot assay (Western blot) and ELISA assay to confirm the assessments of AmGSTF1 antibodies done by Mologic Ltd.
